# Concerning Tuberculin

**Published:** 1913-02-22

**Authors:** 


### The Hospital

The Workers' Newspaper of Administrative Medicine and Institutional Life, Administration, National Insurance and Health.

No. 1390, Vol. LIII. SATURDAY, FEBRUARY 22, 1913. PRICE ONE PENNY.

### CONCERNING TUBERCULIN.

What is quite certain is that we are going to see throughout the country a huge increase in the use of tuberculin. And the reason is very plain; the necessity, to wit, of giving something for his money to the tuberculous contributor to the Insurance Act. If that individual's case is unsuitable for a sanatorium, or if, for fear of losing his situation, he does not want to go there or to stop there long, or if, when discharged thence, he wishes to continue with a course of injections—in all these frequent eventualities he must often have tuberculin administered. The fact is undoubted, and necessarily posits one or two questions. To begin with the most important, Is the tuberculin going to be properly used? For in certain circumstances it may be a dangerous thing. The general practitioner is showing praiseworthy eagerness to learn; at a chance visit to one of the large consumption hospitals a class taking out a course of instruction (at two guineas a head) was found to number about fifty.

Unfortunately, it may be doubted whether any Englishmen, except two, are authorities on the Continental intensive system of using tuberculin. These two are Dr. Egbert Morland and Dr. Camac Wilkinson. The first named, known already by his translation of Bandelier and Roepke's work and by his collaboration in the little (but by no means superficial) publication in the Oxford Medical Series, would do much good if he were at this moment in London demonstrating the "method of Koch," as applied to ambulant treatment. To let one's patient in for a severe constitutional reaction, with consequent loss of a couple of days' work, for want of eliciting the fact that the last injection gave him a rather stiff shoulder, is a blunder which on a large scale would be serious. In the present circumstances then—those of a method originating in Germany and new to this country, about which native leaders of the profession necessarily entertain hazy or mistrustful ideas, but which is bound to be largely used in the near future—it would surely be well if at first ambulant treatment were mainly undertaken by institutions *ad hoc*—namely, tuberculosis dispensaries.

The cases of many consumptives involve at one time or another inter-correspondence of sanatorium representatives, municipal authorities, Boards of Guardians, charitable agencies, firms of employers, and so forth—and centralisation helps all this. Centralisation, again, promotes economy, so important in all anti-tuberculosis measures. The doctor's time will be saved, and a so-to-say whole-time practitioner will be enabled to dilute his own tuberculin, and thus save the use of expensive ready put-up capsules from some wholesale chemist, which is the form the general practitioner would probably employ. Hence a query in passing. The commercial benefits, the trade profits created by medical research—are these to go, lock, stock, and barrel, into the hands of private lay manufacturers? Is the money resulting as a by-product of Koch's brain activity to go to confer that very important powers—namely, money-spending power—upon whoever it may be? The ordinary inventor is protected; not so the medical inventor—as witness an instance, in a small way, in connection with this same subject of tuberculosis. In the early days of anti-tuberculosis effort a medical man devised a handy form of cheap spitting flask and sent it to a manufacturer with an inquiry as to cost of production. The terms were too high and he went to a smaller firm. Very shortly afterwards the first party came out with this very pattern, selling it largely, but never, of course, paying a penny of royalty either to the inventor or to the institution with which he was connected. Now a little tithing of commercial beneficiaries would endow medical research, and provide memorials to Lord Lister, and give the medical profession its proper share of influence without any skimping at all. Returning to the subject of tuberculin, there is an advantage of the dispensary plan which should not, in this Journal, have been left to the last, and that is the favourable mental influence on the patient of the *genius loci*, of the institutional spirit. As to results, if it be asked what may be expected of tuberculin, the reply must be that time alone can show. Meanwhile, a thing which would probably strike any level-headed layman, especially if an institutional worker, is the number of different kinds or preparations of tuberculin in use. There are tuberculins endo-toxic or exo-toxic; albumen free, albumose free; kinds mixed with chemicals, furred with moulds, or made of previously "sensibilised" bacilli, which, too, may be in origin human, bovine, avian, or even vegetable—all of these in varying dosage. About the last new production is the scheme of Much and Leschke, who argue that the anti-bodies in tuberculosis are not simple, but, on the contrary, highly complex, being made up of anti-bodies—partial anti-bodies—to the albuminous, the fatty, and the toxic ingredients of the bacillus of Koch. In every case of tuberculosis there must be made an elaborate examination by means of the methods of complement fixation and hypersensibility, to see which of these anti-bodies is lacking, and, therefore, which particular antigen is indicated.

All this shows, certainly enough, the activity of the restless human spirit striving to satisfy itself. But it also shows the improbability of much therapeutic result having so far been obtained. A short time back we enumerated four confidently recommended remedies for consumption, remarking that it would seem unlikely that all of them would come up to the claims made. Already two of the four have practically dropped out of count. Tuberculin (intensively) probably stands in a different category, but we must wait to see whether its present advocates must be reckoned with the long, long roll of false prophets and unreliable observers to be found in the history of the treatment of consumption. Up to now, and with all its faults, the only landmark is the sanatorium, because the sanatorium is the only method of treatment which has lived to be fifteen years old.

